# Case Report: Ultrasound features of pathological complete response in middle and low rectal cancer with high microsatellite instability after neoadjuvant immunotherapy: a case series

**DOI:** 10.3389/fimmu.2026.1772953

**Published:** 2026-04-13

**Authors:** Li Liang, Qi Wang, Yifeng Chen, Xinhua Zhang, Zubang Zhou, Fang Nie

**Affiliations:** 1 Ultrasound Medical Center, The Second Hospital and Clinical Medical School, Lanzhou University, Lanzhou, China; 2 Department of Ultrasound, Gansu Provincial Hospital, Lanzhou, China; 3 Department of Anorectal Surgery, Gansu Provincial Hospital, Lanzhou, China; 4 Gansu Province Clinical Research Center for Ultrasonography, Lanzhou, China

**Keywords:** immunotherapy, microsatellite instability-high, pathologic complete response, rectal cancer, ultrasound

## Abstract

Accurate assessment of complete clinical response following neoadjuvant chemoradiotherapy remains challenging, and radiological evaluation criteria established for this treatment modality may not be applicable to neoadjuvant immunotherapy (NIT). Currently, there are no published studies describing the ultrasound features of pathological complete response (pCR) in patients with microsatellite instability-high (MSI-H) rectal cancer following NIT. In this case series, we comparatively analyzed multimodal transrectal ultrasound (TRUS) features before and after NIT in three patients with MSI-H mid- to low-rectal cancer. Despite imaging findings suggestive of residual lesions, two patients achieved pCR as confirmed by postoperative pathology, and one patient had no definite atypical components identified on ultrasound-guided biopsy. Our preliminary observations suggest that multimodal TRUS features, including reduced lesion size, diminished vascularity, heterogeneous hypoenhancement with non-enhancing areas on contrast-enhanced ultrasound, and reduced stiffness on shear wave elastography, may serve as potential imaging indicators of pCR in patients with MSI-H mid- to low-rectal cancer. Ultrasound-guided biopsy may provide complementary diagnostic information for confirming pCR in cases with persistent imaging abnormalities.

## 1 Introduction

Colorectal cancer has high incidence and mortality rates (1), with rectal cancer accounting for nearly one-third of all colorectal cancers (2). Only 2–3% of rectal cancers exhibit deficient mismatch repair (dMMR) and/or microsatellite instability-high (MSI-H) status (3), which are associated with a poor response to neoadjuvant chemotherapy (4). However, dMMR/MSI-H serves as a reliable biomarker for neoadjuvant immunotherapy (NIT) in rectal cancer, which has consequently emerged as a promising treatment for patients with dMMR/MSI-H rectal cancer (5, 6). NIT has significantly increased the pathological complete response (pCR) rates in dMMR/MSI-H rectal cancers (7–11). For patients who achieve clinical complete response (cCR) after therapy, the watch-and-wait (W&W) strategy has emerged as an alternative to surgery (5).

Notably, accurate assessment of cCR after NIT is a clinical challenge. Traditional cCR evaluation criteria were developed for neoadjuvant chemoradiotherapy, with the primary criterion being absence of any residual tumor in the primary site (6). Furthermore, traditional cCR evaluation criteria for imaging may not suitable be for NIT (12, 13). Few studies have reported multimodal transrectal ultrasound (TRUS) for assessing the efficacy of neoadjuvant chemoradiotherapy (14–17). However, no relevant reports have been published on the ultrasound features of pCR in patients with MSI-H rectal cancer after NIT. In this study, we present the ultrasound images of three patients with MSI-H rectal cancer located in the middle and lower rectum, who achieved pCR after NIT (sintilimab monotherapy), including the pre- and post-treatment comparisons. Special emphasis is placed on multimodal ultrasound findings of residual lesions following NIT. The study aimed to identify the ultrasound features indicative of pCR, thereby enabling accurate, non-invasive detection of pCR and guiding treatment management in patients with MSI-H rectal cancer.

## 2 Case description

We evaluated three patients who were diagnosed with moderately differentiated rectal adenocarcinoma via colonoscopy biopsy, with MSI-H status. Each patient underwent digital rectal examination (DRE), TRUS, serum tumor markers (including carcinoembryonic antigen, carbohydrate antigen 19-9, carbohydrate antigen 72-4, carbohydrate antigen 242, and carbohydrate antigen 50), computed tomography (CT) and/or magnetic resonance imaging (MRI) during the initial assessment. All patients received neoadjuvant sintilimab monotherapy (200 mg by intravenous infusion, Innovent Biologics, Suzhou, China) every 21 days. Post-treatment response was assessed via the same modalities, with residual lesions precluding cCR determination. Two patients achieved pCR after surgery, while one opted for W&W following ultrasound-guided biopsy negative for residual tumor. **Table 1** summarizes demographics, treatment, imaging, and outcomes.

**Table 1:** Case summary.

Case	Gender/Age (years)	Location	Baseline TM	Baseline TNM (CT/MRI)	Initial TRUS: modalities and features	Preoperative NIT cycles	Preoperative TM	Preoperative TNM (CT/MRI)	Preoperative TRUS: modalities and features	Weeks from last NIT to surgery/biology	Subsequent NIT cycles after surgery/biology	Weeks of follow-up after surgery/biology
1	Female/65	Middle-low rectum	Negative	CT: T3bN1Mx	2D, CDFI, SWE, CEUS irregular lesion with mesorectal invasion, abundant vascularity, hyper-enhancement, and high stiffness	5	Negative	CT: T3aN0Mx	2D, CDFI, SWE, CEUS obvious size reduction, reduced invasion, decreased vascularity, hypoenhancement, and Emean/Emax similar to normal rectal wall.	3	3	17
2	Male/68	Low rectum	CA72-4: 15 U/mL; reference range <12.1 U/mL) Other: negative	MRI/CT: T3cN2Mx	2D, CDFI regular lesion with deep mesorectal invasion and moderate vascularity	4	Negative	mrTRG 2 CT: No obvious change	2D, CDFI, SWE, CEUS slight size reduction, reduced invasion, minimal vascularity, hypoenhancement with large non-enhancing areas, Emean/Emax > normal rectal wall.	8	3	33
3	Female/51	Low rectum	CA72-4:12.4 U/mL; (reference range <10 U/mL) Other: negative	MRI: T2N1Mx	2D, CDFI regular lesion with perilesional vascularity	9	Negative	mrTRG 2	2D, CDFI, SWE, CEUS slight size reduction, absent vascularity, hypoenhancement with large non-enhancing areas, and Emean similar to normal rectal wall.	5	2	30

TM, tumor markers (including carcinoembryonic antigen, carbohydrate antigen 19-9, carbohydrate antigen 72-4, carbohydrate antigen 242, and carbohydrate antigen 50); CA72-4, carbohydrate antigen 72-4; TNM, Tumor-Node-Metastasis; CT, computed tomography; MRI, magnetic resonance imaging; TRUS, transrectal ultrasound; 2D, two-dimensional ultrasound; CDFI, color Doppler flow imaging; SWE, shear wave elastography; CEUS, contrast-enhanced ultrasound; NIT, neoadjuvant immunotherapy; mrTRG, magnetic resonance tumor regression grade; Emean, mean elasticity; Emax, maximum elasticity.

TRUS Protocol: Multimodal TRUS, including two-dimensional ultrasound (2D), color Doppler flow imaging (CDFI), shear wave elastography (SWE) and contrast-enhanced ultrasound (CEUS) were performed by two ultrasound physicians with more than 5 years of experience using a dual-plane transducer. Following 2D, CDFI, and SWE, 2.4 mL of sulfur hexafluoride microbubble contrast agent (SonoVue Bracco, Italy) was injected into the median cubital vein, and contrast videos were recorded for approximately 2 minutes. The detailed TRUS Protocol is provided in **Supplementary Material**.

### 2.1 Case 1

**Figure 1:**
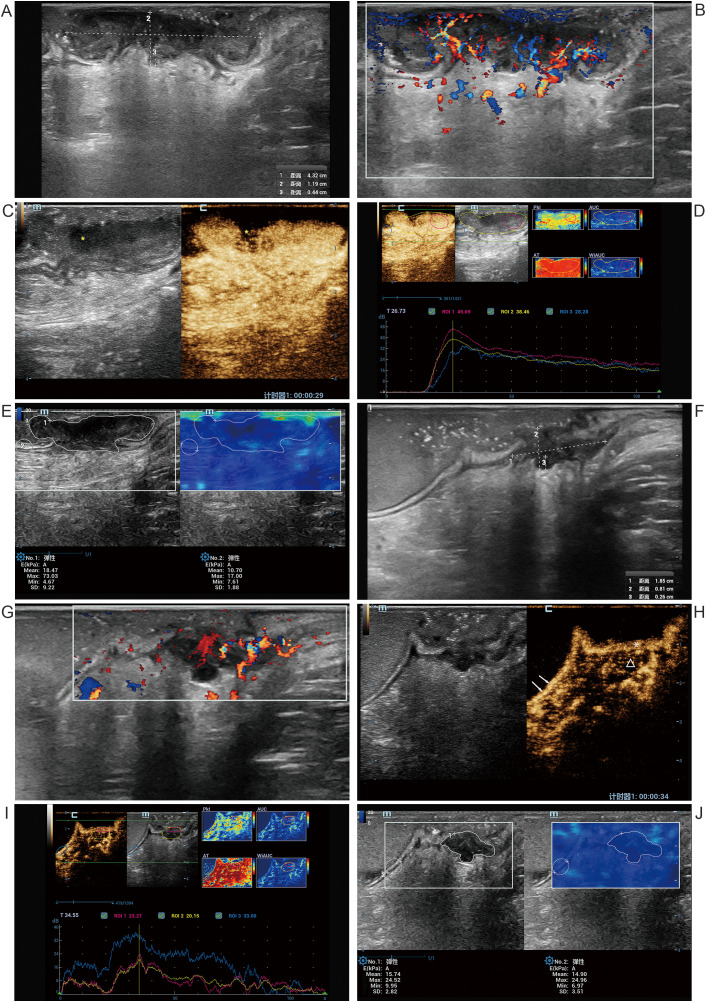
Ultrasound images of a 65-year-old female with MSI-H rectal cancer. Pre-NIT **(A–E)**: **(A)** Sagittal 2D ultrasound shows an irregular hypoechoic lesion (longitudinal length: 43 mm, thickness: 12 mm) with mesorectal invasion of 4 mm. **(B)** CDFI reveals abundant intralesional vascularity. **(C, D)** CEUS demonstrates homogeneous hyper-enhancement (red) compared with adjacent normal rectal wall (blue), with a central non-enhancing area (asterisk). **(E)** SWE shows tumor Emean 18 kPa versus normal rectal wall 11 kPa and Emax 73 kPa, markedly higher than normal wall. Post-NIT **(F–J)**: **(F)** Sagittal 2D ultrasound shows marked lesion size reduction (longitudinal length: 19 mm, thickness: 8 mm) with mesorectal invasion depth decreased to 3 mm. **(G)** CDFI reveals abundant blood flow near the lumen but absent near the mesentery. **(H)** CEUS shows uniform enhancement (asterisk) in the lesion near the lumen, continuous with the normal submucosa (arrow), while the lesion locate in the muscular layer and mesentery exhibits hypoenhancement (triangle). **(I)** TIC analysis shows lower peak intensity of the residual lesion than surrounding normal rectal wall. **(J)** SWE demonstrates lesion Emean and Emax comparable to normal rectal wall. MSI-H, microsatellite instability-high; NIT, neoadjuvant immunotherapy; 2D, two-dimensional ultrasound; CDFI, color Doppler flow imaging; SWE, shear wave elastography; CEUS, contrast-enhanced ultrasound; Emean, mean elasticity; Emax, maximum elasticity; TIC, time-intensity curve.

A 65-year-old female patient with a 1-year history of intermittent, small-volume hematochezia. One month prior to admission, she experienced worsening hematochezia. She underwent left hip arthroplasty 2 years ago, with no family history of genetic disorders. Colonoscopy revealed a mass in the middle-low segment of the rectum. Colonoscopy biopsy confirmed moderately differentiated adenocarcinoma with MSI-H. Serum tumor markers were within normal limits. CT revealed T3bN1Mx stage. Despite passing initial MRI safety screening due to prior arthroplasty, the patient’s MRI was aborted because pelvic susceptibility artifacts impaired rectal imaging quality. Initial ultrasonography revealed an irregular hypoechoic lesion with mesorectal invasion (**Figure 1A**) and abundant intralesional vascularity (**Figure 1B**). CEUS demonstrated homogeneous hyper-enhancement in most regions of the lesion compared with the adjacent normal rectal wall, with a centrally localized non-enhancing area (**Figures 1C, D**). SWE measurements showed that the mean elasticity (Emean) and maximum elasticity (Emax) of the tumor were higher than those of the surrounding normal rectal wall, with Emax markedly exceeding that of the normal rectal wall (**Figure 1E**).

Over the following 4 months, the patient underwent five cycles of NIT. Three weeks after the end of the last cycle, post-NIT assessments were conducted. All assessments were completed within 2 days. Serum tumor markers remained within normal limits. CT scan revealed T3aN0Mx staging, with marked tumor shrinkage compared to pre-NIT.

Post-NIT TRUS revealed that compared to pre-NIT, the lesion’s longitudinal diameter and thickness exhibited marked reductions, mesorectal invasion depth decreased to 3 mm (**Figure 1F**), re-staging remained at T3b. CDFI revealed abundant vascularity in the lesion adjacent to the intestinal lumen, whereas no flow signals were detected in the deeper portion near the mesentery (**Figure 1G**). CEUS revealed that the surface enhancement pattern of the residual lesion was comparable to that of the adjacent normal intestinal mucosa, with heterogeneous hypoenhancement in muscularis propria and mesenteric components (**Figure 1H**). Quantitative CEUS analysis revealed baseline fluctuations due to premature contrast administration before protocol initiation from suboptimal venous access integrity. Quantitative CEUS analysis revealed that the peak intensity of the residual lesion is lower than that of the surrounding normal rectal wall (**Figure 1I**). Emean and Emax values of the lesion were comparable to adjacent normal rectal wall (**Figure 1J**). The comprehensive assessment indicates good response to the NIT.

Two days after post-NIT TRUS, the patient underwent laparoscopic anterior rectal resection with ileostomy. Postoperative histopathology confirmed pCR, corresponding to tumor regression grade 0 (**Supplementary Figure 1**). Tumor regression grade followed the 7th edition of the American Joint Committee on Cancer (AJCC) staging system (18).

The patient received three subsequent cycles of NIT postoperatively. At 17 weeks after surgery, DRE, TRUS, serum tumor markers, and CT exhibited no abnormalities.

### 2.2 Case 2

A 68-year-old man presented with 2-month painless hematochezia, frequent bowel movements, and no family history of genetic disorder. DRE revealed hard lower rectal mass with blood-stained finger cot and colonoscopy biopsy confirmed moderately differentiated adenocarcinoma with MSI-H. Carbohydrate antigen 72-4 was elevated (15.3 U/mL; reference range <10 U/mL).

**Figure 2:**
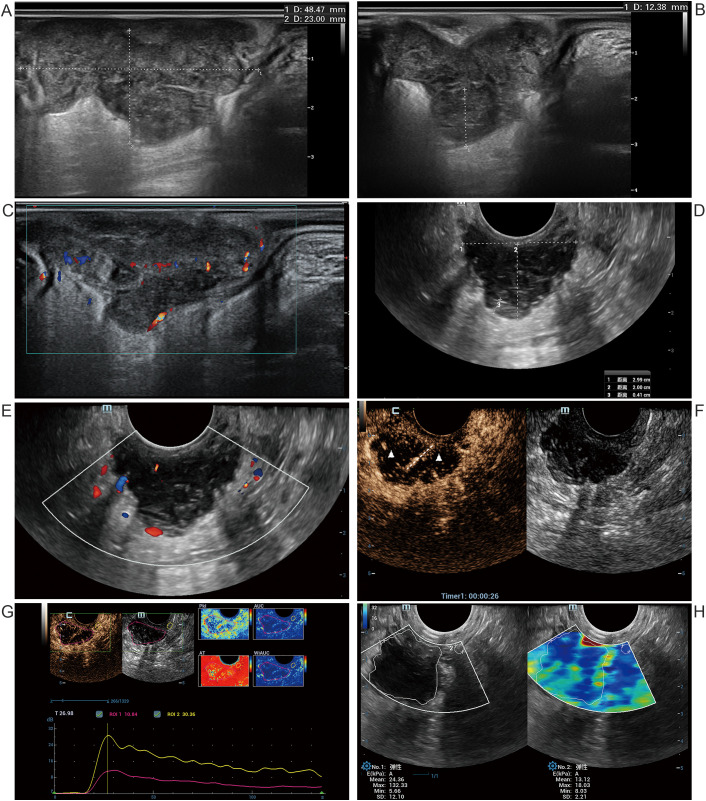
Ultrasound images of a 68-year-old male with MSI-H rectal cancer. Pre-NIT **(A–C)**: **(A)** Sagittal 2D ultrasound shows a lower rectal hypoechoic lesion (longitudinal length:48 mm, thickness:23 mm). **(B)** Mesorectal invasion depth is 12 mm. **(C)** CDFI demonstrates moderate intralesional vascularity. Post-NIT **(D–H)**: **(D)** Transverse 2D ultrasound reveals tumor thickness 20 mm, invasion depth 4 mm, and transverse diameter 30 mm. **(E)** CDFI shows minimal vascularity. **(F)** CEUS demonstrates linear enhancement (dotted line) with overall heterogeneous hypoenhancement and extensive non-enhancing regions (triangle). **(G)** TIC analysis shows significantly lower peak enhancement in the lesion (red) compared with that in the normal rectal wall (yellow). **(H)** SWE shows lesion Emean of 24 kPa in the lesion versus 13 kPa in the normal rectal wall and an Emax of 132 kPa versus 18 kPa, respectively. MSI-H, microsatellite instability-high; NIT, neoadjuvant immunotherapy; 2D, two-dimensional ultrasound; CDFI, color Doppler flow imaging; CEUS, contrast-enhanced ultrasound; TIC, time-intensity curve; SWE, shear wave elastography; Emean, mean elasticity; Emax, maximum elasticity.

Initial TRUS revealed lower rectal hypoechoic lesion with well-defined margins, lobulated shape (**Figure 2A**), maximal mesorectal invasion depth 12 mm (**Figure 2B**), consistent with T3c stage. CDFI showed moderate intralesional vascularity (**Figure 2C**); MRI showed mixed cystic-solid lesion, T3cN2Mx stage.

Over the following five months, the patient underwent four cycles of NIT. Seven weeks after the end of the last cycle, post-NIT assessments were conducted. All assessments were completed within two days. Serum tumor markers within normal limits. DRE findings were unchanged from pre-NIT. MRI revealed a slight reduction in tumor size compared with pre-NIT imaging, along with extensive mucinous components within the lesion, with magnetic resonance tumor regression grade (mrTRG) assigned as 2.

Intestinal segment stenosis prevented passage of the dual-plane linear array probe, precluding use of the same pretreatment sagittal plane for evaluation. Post-NIT TRUS showed that, compared to pre-NIT, the lesion’s mesorectal invasion depth decreased to 4 mm, thickness had slight reductions, transverse diameter was 30 mm (**Figure 2D**), re-staging as T3b. CDFI revealed minimal vascularity (**Figure 2E**). CEUS revealed linear enhancement within heterogeneous hypoenhancement and large non-enhancing areas (**Figures 2F, G**). The Emean and Emax of the residual lesion were higher than those of adjacent normal rectal wall, with more pronounced Emax difference (**Figure 2H**). The above assessment cannot determine whether the treatment response is good.

Two days after post-NIT assessment, the patient underwent laparoscopic abdominoperineal resection. Postoperative pathology confirmed pCR (**Supplementary Figure 2**).

The patient received two subsequent postoperative NIT cycles. At 33 weeks postoperatively, serum tumor markers and MRI showed no abnormalities.

### 2.3 Case 3

A 51-year-old female patient presented with 1-year hematochezia, new onset of post-defecation incomplete evacuation 1 week before admission, and no significant medical or family history. DRE revealed hard lower rectal mass with bloody finger cot. Colonoscopy biopsy confirmed moderately differentiated adenocarcinoma with MSI-H. Carbohydrate antigen 72-4 was elevated (12.4 U/mL; reference range <10 U/mL). MRI revealed T2N1Mx staging (**Supplementary Figure 3**).

**Figure 3:**
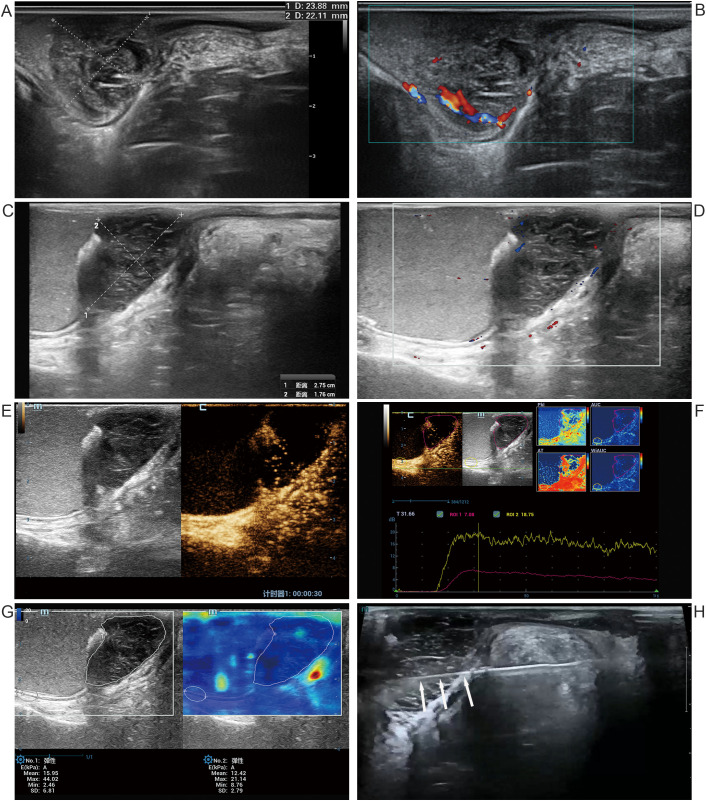
Ultrasound images of a 51-year-old female with MSI-H rectal cancer. Pre-NIT **(A, B)**: **(A)** Sagittal 2D ultrasound shows a regular hypoechoic lesion (longitudinal length: 24 mm, thickness: 22 mm). **(B)** CDFI reveals peripheral lesion vascularity. Post-NIT **(C–H)**: **(C)** Sagittal 2D shows a regular hypoechoic residual lesion (longitudinal length: 28 mm, thickness: 18 mm). **(D)** CDFI shows the disappearance of blood flow signals. **(E)** CEUS demonstrates hypoenhancement with internal heterogeneous punctate enhancement and large non-enhancing areas. **(F)** TIC analysis shows lower peak intensity in the lesion (red) than in the normal wall (yellow). **(G)** SWE shows lesion Emean of 16 kPa in the lesion versus 12 kPa in the normal rectal wall and an Emax of 44 kPa versus 21 kPa, respectively. **(H)** Ultrasound-guided biopsy shows accurate needle (arrow) placement. MSI-H, microsatellite instability-high; NIT, neoadjuvant immunotherapy; 2D, two-dimensional ultrasound; CDFI, color Doppler flow imaging; CEUS, contrast-enhanced ultrasound; TIC, time-intensity curve; SWE, shear wave elastography; Emean, mean elasticity; Emax, maximum elasticity.

Initial TRUS revealed a well-defined lower rectal hypoechoic mass adjacent to the anorectal ring, confined to the muscularis propria with T2 stage (**Figure 3A**), and peripheral lesion vascularity (**Figure 3B**).

Given the tumor’s proximity to the anorectal ring and the high risk of irreversible sphincter injury with permanent loss of anal function, the patient declined surgery. Due to the patient’s strong preference for anus preservation and biopsy-confirmed MSI-H status, NIT was initiated 1 week later. Over the following eight months, the patient underwent nine cycles of NIT.

Five weeks after the end of the last cycle, post-NIT assessments were conducted. All assessments were completed within 2 days. Serum tumor markers were within normal limits. DRE findings were unchanged from pre-NIT. MRI revealed a slight reduction in tumor size compared with pre-NIT imaging, along with extensive mucinous components within the lesion (**Supplementary Figure 3**).

Post-NIT TRUS showed that, compared to pre-NIT, the lesion’s thickness decreased from 22 mm to 18 mm, but its longitudinal diameter increased from 24 mm to 28 mm (**Figure 3C**), no significant intratumoral vascularity (**Figure 3D**). CEUS showed heterogeneous hypoenhancement of the residual lesion, with internal heterogeneous punctate enhancement and large non-enhancing areas (**Figures 3E, F**). Emean of the residual lesion was comparable to adjacent normal rectal wall, Emax was higher than adjacent normal rectal wall (**Figure 3G**).

A formal multidisciplinary team (MDT) meeting was convened, comprising colorectal surgeons, medical oncologists, radiologists, and ultrasound physicians. The MDT conducted a comprehensive review of all available imaging data and found that this patient’s imaging findings (2D, CEUS, MRI) after NIT were highly similar to those of Case 2 in our case series. Specifically, both Case 2 and Case 3 demonstrated a slight reduction in lesion size; CEUS revealed heterogeneous hypoenhancement with large non-enhancing areas within the residual lesions, whereas MRI showed abundant mucinous components within the residual lesions. Notably, Case 2 was confirmed to have achieved pCR through surgical resection. Based on the similarity of the imaging features between the two cases and this patient’s strong desire to preserve the anus, the MDT considered the possibility of pCR in this patient and recommended histopathological confirmation. Ultrasound-guided targeted core biopsy was selected over endoscopic biopsy for the following reasons: CEUS revealed largely avascular regions within the residual lesion; therefore, real-time ultrasound guidance with vascular imaging enabled selective sampling of vascularized tumor tissue while avoiding non-viable areas (including mucin pools, necrotic tissue, and post-treatment fibrosis), thereby optimizing diagnostic yield. Following assessment by an interventional sonographer with 10 years of experience in ultrasound-guided procedures, the transperineal approach was considered to provide the optimal needle trajectory for this low rectal lesion, ensuring precise targeting of contrast-enhancing regions. Consequently, ultrasound-guided transperineal biopsy was performed for Case 3 on the day after post-NIT TRUS. The biopsy needle was accurately positioned within the contrast-enhancing area of the residual lesion (**Figure 3H**).

The biopsy pathology revealed no definite atypical components. Based on these results, the MDT conducted an in-depth discussion of the risks and benefits of active surveillance versus immediate surgery. Considering the patient’s strong desire for anal preservation, the MDT and patient jointly decided to adopt a W&W strategy, with a recommendation for close clinical monitoring.

The patient received two subsequent cycles of NIT after ultrasound-guided biopsy. The interval between biopsy and the last phone follow-up was 30 weeks. During this period, the patient reported no discomfort and did not undergo any imaging examinations.

## 3 Discussion

To our knowledge, this is the first report describing the ultrasound features of pCR in patients with MSI-H rectal cancer after NIT. Organ preservation is crucial for patients with rectal cancer, particularly for middle-low rectal cancer. Neoadjuvant chemotherapy cannot be recommended for dMMR (6). For patients with dMMR/MSI-H locally advanced or mid-low rectal cancer, neoadjuvant immunotherapy can be the preferred option (5, 6). PD-1 monotherapy has been associated with high cCR (75–100%) and pCR (47.6–75.9%) rates, with organ preservation achieved in up to 100% of patients (11). The monotherapy with PD-1 inhibitors not only shows remarkable efficacy but also has sufficient safety (19). For patients who have achieved cCR following neoadjuvant therapy, they can be managed by the W&W strategy (5). Therefore, assessing cCR following NIT in dMMR/MSI-H has emerged as an important clinical focus, guiding treatment strategies and aiding in the prediction of patient prognoses.

The European Society for Medical Oncology (ESMO) guideline was used to evaluate cCR in this study (6). The current criteria for evaluating cCR are based on DRE, endoscopy, and MRI. Under this consensus, cCR is defined as: 1. normal DRE, 2. no visible lesion at endoscopy, 3. no residual tumor or residual fibrosis only, and draining lymph nodes on imaging with MRI. We observed an interesting phenomenon: in all three patients, the imaging examinations (MRI/CT and TRUS) performed before surgery/biopsy revealed the presence of residual lesions that did not conform to the current criteria for evaluating cCR. This may be explained by immunotherapy-induced histological changes in dMMR/MSI-H rectal cancer—such as lymphocyte infiltration, necrosis, fibrosis, and edema—which may lead to pseudoprogression and false-positive results on imaging (12, 13).

Accurate preoperative identification of cCR following neoadjuvant therapy is critical for rectal cancer treatment decisions. While MRI remains the cornerstone for rectal cancer staging, its post-neoadjuvant diagnostic performance and clinical utility are controversial (20, 21). The mrTRG was developed to predict histopathological response to chemoradiotherapy in locally advanced rectal cancer (22). The mrTRG is a standardized system for assessing the proportion of residual viable tumor relative to treatment-induced changes (including fibrosis, edema, inflammatory infiltration, and necrosis) on post-treatment MRI (23). The mrTRG demonstrates limited accuracy in predicting pCR after neoadjuvant therapy, although it has been shown to correlate with the degree of tumor regression during treatment (24). Notably, mrTRG 1 has high specificity for pCR but suboptimal sensitivity, thereby limiting its utility as a criterion for selecting less aggressive treatment strategies after neoadjuvant therapy (25). cCR on MRI is defined by homogeneous low T2-weighted signal intensity in the tumor bed, no intermediate-signal tumor tissue, and resolved restricted diffusion on diffusion-weighted imaging (23). However, it is noteworthy to mention that although all three reported cases achieved pCR, the imaging findings were inconsistent with the pathological outcomes. Both MRI and TRUS showed residual rectal lesions that failed to meet MRI criteria for cCR. In Case 2, the patient was classified as mrTRG 2 after NIT, while mrTRG 1 corresponds to pCR (22). Another study revealed that neither rectal MRI nor endorectal ultrasound (performed by endoscopists) provided a sufficiently accurate assessment of cCR in patients with rectal cancer following neoadjuvant therapy, particularly NIT, and that cCR did not reliably predict pCR (26).

However, no validated standard equivalent to mrTRG exists for evaluating tumor regression based on ultrasound features after neoadjuvant therapy. Therefore, it remains a challenge for sonographers to accurately assess therapeutic efficacy using ultrasound images after neoadjuvant therapy, especially for patients with MSI-H rectal cancer, to determine whether they have achieved pCR after NIT. Multimodal TRUS may be necessary to more accurately evaluate the efficacy of neoadjuvant therapy, such as CEUS, SWE.

Comparison of the 2D images obtained before and after immunotherapy showed that the lesion in Case 1 decreased significantly in size, while the lesions in Cases 2 and 3 showed no significant reduction. Infiltration depth decreased in Cases 1 and 2. Longitudinal diameter and thickness diameter of the lesion decreased in the good response group (14). CDFI showed a marked reduction in tumor vascularity in Case 1; however, in Cases 2 and 3, it was less obvious. This discrepancy may be attributed to the limited tumor blood supply at baseline, or it could result from the use of different ultrasound diagnostic systems before and after treatment, as these systems may possess inherent differences in the sensitivity of color flow detection. However, the evaluation value of 2D imaging and CDFI for the efficacy of neoadjuvant therapy is rather limited, and it is difficult to distinguish residual lesions from fibrosis and edema after treatment.

Time-intensity curve parameters from CEUS are correlated with pathological prognostic factors, including TN stage, lymphovascular invasion, perineural invasion, and tumor differentiation in rectal adenocarcinomas (27). In Case 1, pre-NIT CEUS revealed homogeneous hyper-enhancement with early wash-in, which changed to heterogeneous hypoenhancement after NIT. This pattern aligns with a prior study reporting that downgrades in CEUS inhomogeneity grade significantly differed between the good and poor response group (14, 28). Across all three Cases, CEUS demonstrated heterogeneous hypoenhancement, consistent with findings that the complete response group had significantly lower peak intensity compared with the non-response group (17). Notably, a retrospective analysis of Cases 2 and 3 revealed that the non-enhancing areas on CEUS corresponded to large mucinous lakes identified on pathological sections. Collectively, these findings indicate that CEUS may serve as a valuable tool for evaluating tumor response after NIT.

SWE assessment in Case 1 revealed that the tumor was initially harder than the surrounding normal rectal wall before NIT. Both the Emean and Emax elasticity values of the tumor decreased after treatment, with a marked reduction in Emax, and the post-NIT Emean and Emax values of tumor approximated to the surrounding normal rectal wall. These findings align with previous study, reporting that tumor Emean acts as an independent predictor for the diagnosis of a complete response (16). Emean and Emax values were significantly lower in the pCR group than in the non-pCR group (29). Moreover, the Emean and Emax before and after chemoradiotherapy differed significantly between patients who did and did not exhibit tumor downstaging for locally advanced rectal cancer (30). A prospective study revealed that Emean exhibited excellent diagnostic performance and that SWE significantly enhanced the diagnostic accuracy of conventional endorectal ultrasound for ypT0 stage prediction (31). SWE maybe an effective ultrasound technique to evaluate tumor downstaging. In Cases 2 and 3, post-NIT Emean and Emax values of the entire tumor lesion area remained higher than those of the normal rectal wall. Regrettably, pre-treatment SWE data for Cases 2 and 3 were unavailable; therefore, we cannot compare the pre- and post-NIT images.

Transrectal ultrasound-guided true-cut biopsy enhances the diagnostic accuracy of conventional modalities for predicting pCR in patients following neoadjuvant treatment (32). It suggests that when imaging modalities cannot draw an affirmative conclusion, ultrasound-guided biopsy serves as a valuable tool for re-evaluating pCR. Compared with endoscopic biopsy, ultrasound-guided targeted core biopsy may achieve higher diagnostic accuracy for residual rectal lesions by utilizing TRUS guidance to selectively sample vascularized tumor tissue and avoid non-viable areas (such as mucin pools, necrosis, fibrosis), resulting in improved sampling precision. Furthermore, from a pathologically perspective, residual rectal tumor cells following neoadjuvant treatment are predominantly localized in the muscularis propria rather than the mucosa (33), which renders endoscopic forceps biopsy insufficient for obtaining representative tumor tissue (34). In Case 3, ultrasound-guided transperineal biopsy of the rectal mass revealed no atypical cells upon histopathological examination. Because the tumor located in the low rectum, anus-preserving surgery would not have been feasible for this patient. Based on the patient’s biopsy pathology and strong desire to preserve the anus, the patient chose the W&W strategy. The W&W strategy is a nonsurgical management approach (23) used in Cases where patients may be eligible for organ preservation. It avoids unnecessary surgery that would result in the removal of the tumor-free rectum, allows patients with middle- and low-rectal cancer, who account for 88.8% of all rectal cancer cases (35) to preserve the anus, avoid permanent colostomy and surgical complications, and significantly improves a patient’s quality of life (36–38).

With the development of imaging biomarkers, ultrasound-bases models demonstrated superior diagnostic performance compared with clinical models in predicting the efficacy of neoadjuvant therapy in patients with locally advanced rectal cancer (14, 15, 28). A predictive model using image-text features extracted from endorectal ultrasound via contrastive language-image pretraining can predict tumor regression grade before neoadjuvant therapy (39). Further research is required to validate its generalizability, with the hope that it may be applied to clinical settings in the future.

This study has several limitations. First, the extremely small sample size (n=3) limits the generalizability of this study. Second, baseline SWE and CEUS images were unavailable for Cases 2 and 3, limiting the evaluation of treatment-related changes in elastographic and contrast-enhanced parameters. Third, the current follow-up duration is insufficient to evaluate long-term oncologic outcomes, as Case 3, managed with the W&W strategy, has only undergone telephone follow-up, without serial imaging or laboratory data to monitor disease status.

## 4 Conclusion

We report a case series of three patients with MSI-H mid- to low-rectal cancer who achieved pCR following NIT, despite residual radiological abnormalities on post-treatment imaging. Our preliminary observations suggest that multimodal TRUS features may correlate with pCR, including reduced lesion size, decreased intratumoral vascularity, heterogeneous hypoenhancement, non-enhancing areas within residual lesions on CEUS, lower Emean and Emax values on SWE, and Emean values approaching those of the adjacent normal rectal wall. Additionally, ultrasound-guided biopsy may provide complementary information for pCR assessment in this small cohort. Overall, our findings from this case series suggest that multimodal ultrasound may offer potential imaging indicators for distinguishing pCR from residual disease in MSI-H mid- to low-rectal cancer. These observations warrant further validation in larger, prospective studies to determine whether such features can support the safe implementation of a W&W strategy in this patient population.

## Data Availability

The original contributions presented in the study are included in the article/**Supplementary Material**. Further inquiries can be directed to the corresponding author.

## Glossary

dMMR: deficient mismatch repair
MSI-H: microsatellite instability-high
NIT: neoadjuvant immunotherapy
pCR: pathological complete response
cCR: clinical complete response
W&W: watch-and-wait
CT: computed tomography
MRI: magnetic resonance imaging
TRUS: transrectal ultrasound
CEUS: contrast-enhanced ultrasound
SWE: shear wave elastography
Emean: mean elasticity
Emax: maximum elasticity
DRE: digital rectal examination
2D: two-dimensional ultrasound
CDFI: color Doppler flow imaging
mrTRG: magnetic resonance tumor regression grade
MDT: multidisciplinary team
