# Supplementary figures and images for: Case Report: Ultrasound features of pathological complete response in middle and low rectal cancer with high microsatellite instability after neoadjuvant immunotherapy: a case series
Source: Front Immunol. 2026 Apr 13;17:1772953. doi: 10.3389/fimmu.2026.1772953 (PMC13111403; doi:10.3389/fimmu.2026.1772953)

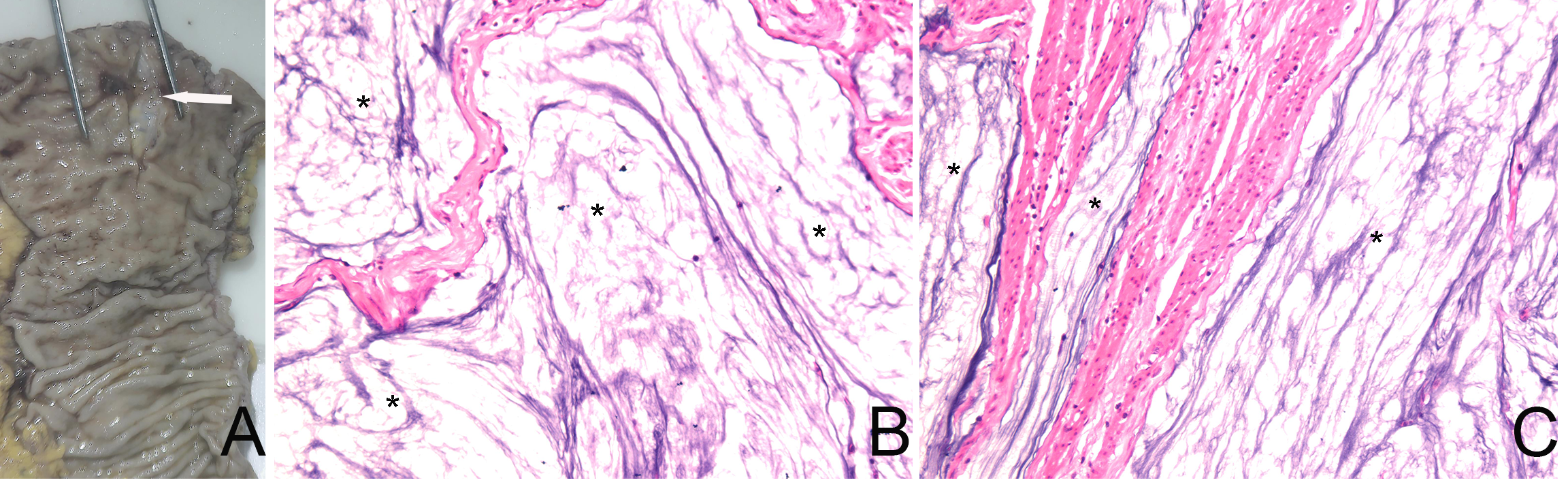

Supplement: Supplementary file 1 [file Image1.tif]

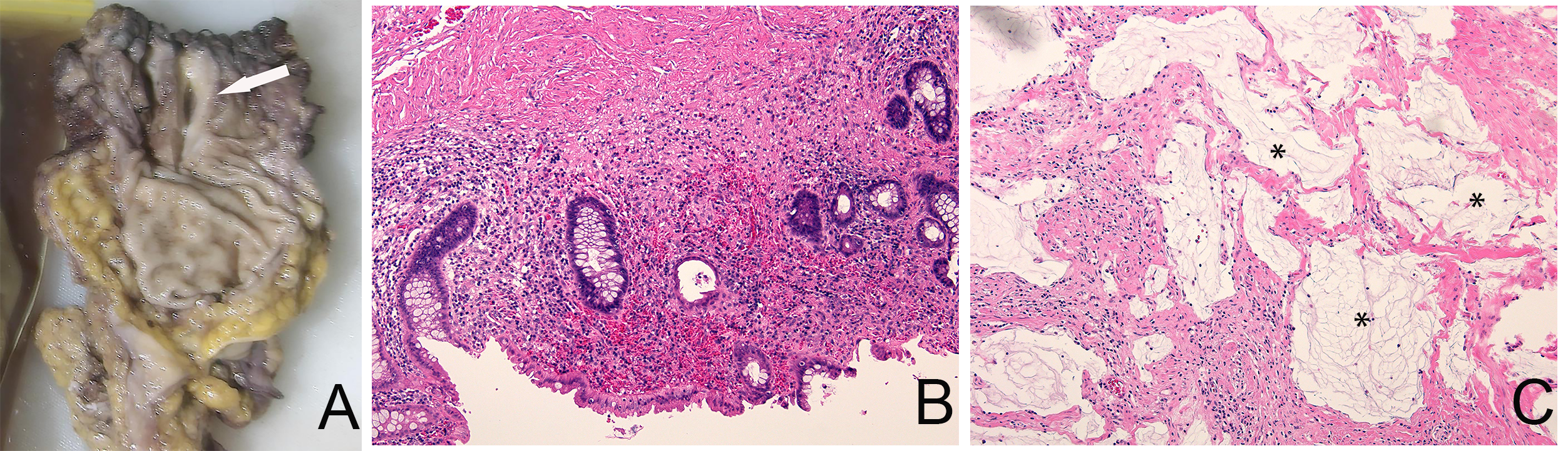

Supplement: Supplementary file 2 [file Image2.tif]

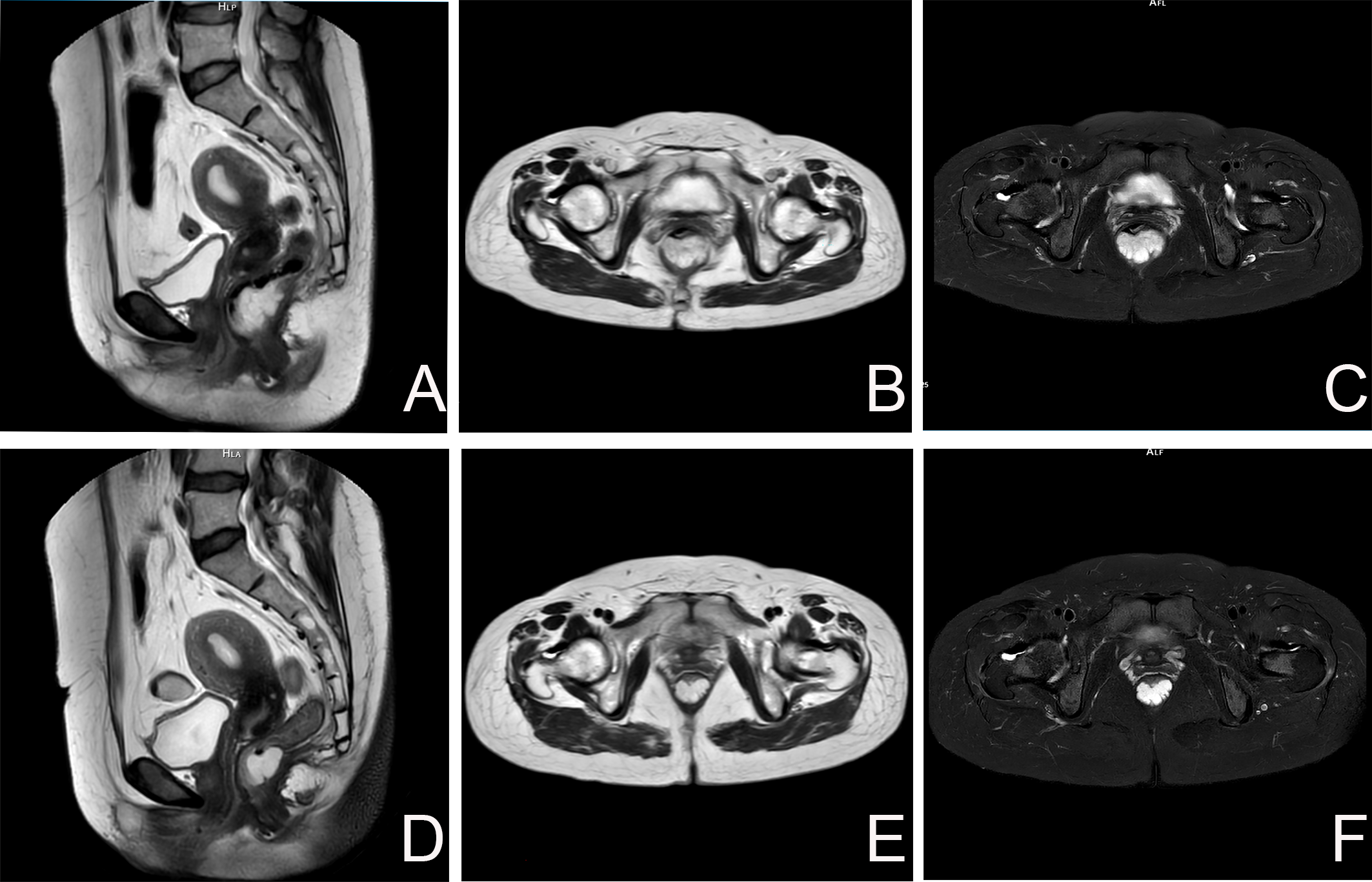

Supplement: Supplementary file 3 [file Image3.tif]
