# Supplementary material for: Case Report: Ultrasound features of pathological complete response in middle and low rectal cancer with high microsatellite instability after neoadjuvant immunotherapy: a case series
Source: Front Immunol. 2026 Apr 13;17:1772953. doi: 10.3389/fimmu.2026.1772953 (PMC13111403; doi:10.3389/fimmu.2026.1772953)
Supplement: Supplementary file 4 [file Table1.docx]

**Supplementary TRUS Protocol:**

This study utilized two dedicated systems for TRUS examinations. For pre-NIT TRUS in Cases 2 and 3, a SonoScape S50 ultrasound system (Shenzhen, China) equipped with a BCL10-5 dual-plane transrectal probe (convex array: 3.5–8.0 MHz, linear array: 7–12 MHz) was used. For pre-NIT TRUS in Case 1 and post-NIT TRUS in all three cases, a Mindray Resona R9 ultrasound system (Shenzhen, China) equipped with an ELC13-4U dual-plane transrectal probe (convex array: 3.5–9.5 MHz, linear array: 3.2–12.8 MHz) was employed.

Contrast-enhanced ultrasound (CEUS) protocol

The ultrasound plane showing the largest tumor area was selected. The CEUS mode was switched on. All CEUS examinations were performed using a low mechanical index (MI) ranging from 0.061 to 0.065, with the frame rate dynamically adjusted between 10 and 13 frames per second based on imaging depth. A 2.4 mL suspension of SonoVue (Bracco, Milan, Italy) was rapidly injected into the patient’s antecubital vein, followed by a 5-mL saline flush to ensure complete contrast delivery. Imaging acquisition was initiated immediately upon contrast administration, with dynamic contrast-enhanced videos continuously recorded and stored for approximately 2 minutes on the Resona R9 ultrasound system workstation.

For quantitative analysis, time-intensity curves and associated parameters were automatically generated via the system’s internal software. The region of interest was manually delineated to encompass the entire tumor lesion. A control region of interest was placed on the entire layer of normal rectal wall at a distance of at least 5 mm from the tumor to provide a reference for comparison. Enhancement patterns were classified as hyperenhancement, isoenhancement or hypoenhancement relative to the adjacent normal rectal wall.

Shear wave elastography (SWE) protocol

The ultrasound section showing the deepest tumor infiltration was selected in Cases 1 and 2. The ultrasound plane showing the largest tumor area was selected in Case 3. The SWE mode was switched on. The SWE examination was initiated when the tumor was clearly visible. B-mode ultrasound and elastography images were displayed simultaneously in dual-display mode. The system settings were standardized, and the maximum elasticity modulus was set at 100 kPa. The sampling frame was adjusted to encompass the entire tumor lesion along with peritumoral tissue. When satisfactory images were obtained, the dynamic video (5–10 s each) was recorded. Three stable images with good color filling were selected from the dynamic video (1). Three SWE images containing the tumor lesion and surrounding normal rectal wall, which were at a distance of at least 5 mm from the tumor, were analyzed.

Quantitative stiffness measurements were performed using the quantification tool provided by the equipment. For tumor lesions, the ROI was manually traced to encircle the entire tumor boundary. For the distant normal rectal wall, the circular ROI covered the entire rectal wall. SWE parameters including maximum (Emax), mean (Emean), minimum (Emin) elasticity, were automatically calculated by the system software. Representative elastography images were selected for presentation. Measurements were not averaged, and intraobserver and interobserver variability were not evaluated in this case report.

**References**

1. Qian Q, Zhuo M, Chen X, Zeng B, Tang Y, Xue E, et al. Shear-wave elastography predicts T-restaging and pathologic complete response of rectal cancer post neoadjuvant chemoradiotherapy. Abdom Radiol (NY). 2024;49(8):2561-73.

**Supplement Figure Legends**

**Supplementary Figure 1.** Pathological findings of Case 1. **(A)** Macroscopic view of the resected rectum showing an ulcerated mass (arrow), with a gray-white, solid appearance. **(B and C)** Microscopic views of the resected rectum showing extensive mucin pool formation (asterisk) throughout the entire rectal wall, with no residual tumor cells identified (H&E staining, ×200).

**Supplementary Figure 2.** Pathological findings of Case 2. **(A)** Macroscopic view of the resected rectum showing an ulcerative-type mass (arrow), with a gray–white and solid appearance. B and C are microscopic views of the resected rectum (H&E staining, ×100). **(B)** No tumor cells are identified in the rectal mucosal layer. **(C)** Extensive mucin pool formation (asterisk), with no residual tumor cells identified.

**Supplementary Figure 3.** MRI images of a 51-year-old female with MSI-H rectal cancer.
(A, D) sagittal T2-weighted images; (B, E) axial T2-weighted images; (C, F) axial T2-weighted fat-suppressed images.
Pre-NIT (A–C): The tumor demonstrates hyperintensity on T2-weighted imaging.
Post-NIT (D-F): The residual lesion shows reduction in tumor size compared with pre-NIT imaging, with abundant mucinous components within the lesion.

Abbreviations: MRI: magnetic resonance imaging, MSI-H, microsatellite instability-high; NIT, neoadjuvant immunotherapy.
